# Gallbladder Ciliated Foregut Cyst Suspected of Malignancy Preoperatively

**DOI:** 10.1155/2021/6222947

**Published:** 2021-10-21

**Authors:** Chikanori Tsutsumi, Toshiya Abe, Hirotaka Kuga, So Nakamura, Kazuyoshi Nishihara, Sadafumi Tamiya, Toru Nakano

**Affiliations:** ^1^Department of Surgery, Kitakyushu Municipal Medical Center, Kitakyushu, Japan; ^2^Department of Pathology, Kitakyushu Municipal Medical Center, Kitakyushu, Japan

## Abstract

**Background:**

Gallbladder ciliated foregut cysts (CFCs) of the lower diaphragm are extremely rare. Furthermore, they are rarely suspected of malignancy preoperatively. *Case Presentation*. A 50-year-old woman was referred to our hospital for further examination and treatment of a gallbladder tumor that was detected using abdominal ultrasonography (US). After a close inspection, she was diagnosed with a gallbladder tumor that was possibly malignant. Accordingly, open whole layer cholecystectomy was performed because intraoperative US revealed a tumor located on the intraperitoneal side of the gallbladder, and a rapid intraoperative pathological diagnosis identified no malignancy. A postoperative pathological examination revealed a cystic lesion with thin walls covered with ciliated epithelium, which laid on a connective tissue with smooth muscle fibers. Based on the above results, the final pathological diagnosis was CFC of the gallbladder without malignancy.

**Conclusions:**

Cases of gallbladder CFC can be considered as cysts requiring treatment owing to CFCs' potential for malignant transformation and high-frequency symptoms.

## 1. Introduction

Ciliated foregut cysts (CFCs) are rare congenital cysts arising from the remnant embryonic foregut and are usually located above the diaphragm [1]. Most CFCs located below the diaphragm are found in the liver. However, CFCs of the lower diaphragm are rarely found in the gallbladder [2, 3]. The biological behavior of these CFCs remains to be clarified because of their rarity. Furthermore, gallbladder CFCs are rarely suspected of malignancy preoperatively. Herein, we report on an extremely rare case of CFC of the gallbladder suspected to be a malignant tumor preoperatively.

## 2. Case Presentation

**Figure 1:**
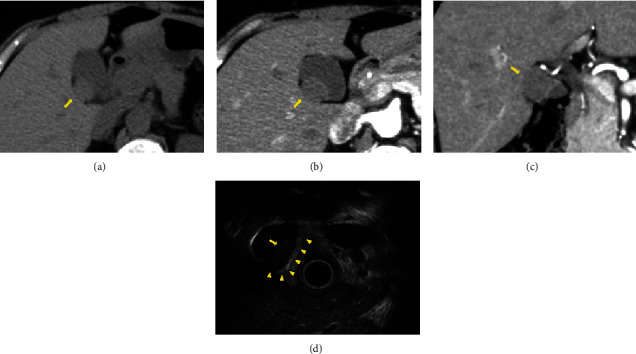
(a) Plain phase of CT showed a low-intensity lesion in the neck of the gallbladder (arrow). (b, c) Arterial phase of the CT revealed a lesion that was contrasted in the neck of the gallbladder, which has an unclear border with the liver (arrow). (d) Endoscopic US showed an isoechoic lesion on the intraperitoneal side of the gallbladder (arrow), without any breakage in the gallbladder wall (arrowhead). CT: computed tomography; US: ultrasonography.

A 50-year-old woman was referred to our hospital for further examination and treatment of a gallbladder tumor detected using abdominal ultrasonography (US). She had no history of malignancy, and physical and laboratory examinations, including tumor marker tests, revealed no specific findings. Contrast-enhanced computed tomography (CT) revealed a 20 mm lesion that was contrasted in the neck of the gallbladder, which had an unclear border with the liver (Figures 1(a)–1(c)). No enlarged lymph nodes or distant metastases were observed. Endoscopic US showed an isoechoic lesion on the intraperitoneal side of the gallbladder without a broken gallbladder wall (Figure 1(d)). Magnetic resonance imaging (MRI) was not performed because the patient was claustrophobic.

**Figure 2:**
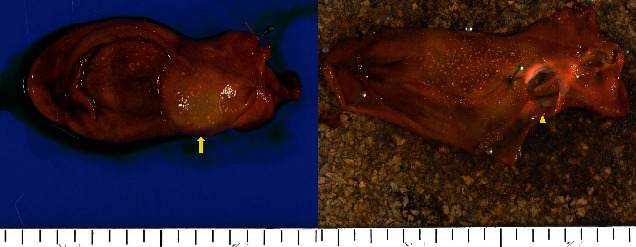
Macroscopic examination of the resected specimen identified gross appearance of a submucosal tumor in the neck of the gallbladder (arrow); the tumor appears to be a unilocular cyst filled with mucoid liquid (arrowhead).

**Figure 3:**
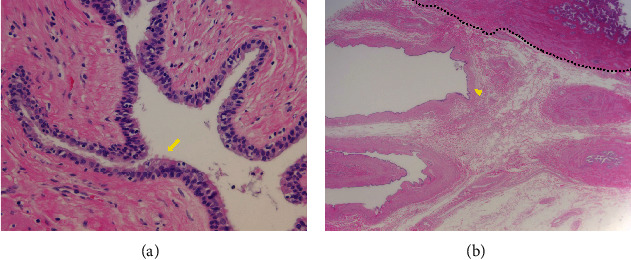
Histopathological specimen (hematoxylin-eosin staining) showed a cystic lesion with thin walls covered with ciliated epithelium, laid on a connective tissue with smooth muscle fibers (arrow), and the cyst (arrowhead) located just outside the gallbladder wall (dotted line). (a) ×20 and (b) ×400 original magnification.

These results suggested that it could be gallbladder cancer. Accordingly, open whole layer cholecystectomy without lymph node dissection and hepatectomy were performed because intraoperative US revealed that the tumor was located on the intraperitoneal side of the gallbladder, and rapid intraoperative pathological diagnosis identified no malignancy. A macroscopic examination of the resected specimen showed a gross appearance of a submucosal tumor in the neck of the gallbladder, and the tumor was found to be a unilocular cyst filled with mucoid liquid (Figures 2(a) and 2(b)). A postoperative pathological examination revealed a cystic lesion with thin walls covered with ciliated epithelium, which was laid on a connective tissue with smooth muscle fibers (Figure 3(a)). It was clearly separated from the gallbladder and located just outside the gallbladder wall without luminal communication (Figure 3(b)). Additionally, the lesion did not show any malignant findings. Based on the above results, the final pathological diagnosis was gallbladder CFC. Her postoperative course was uneventful, and the patient was discharged in a good condition.

## 3. Discussion

CFCs are congenital lesions that develop in the anterior primitive intestine. They are usually found above the diaphragm as cysts on the bronchi or esophagus [1]. Below the diaphragm, they are generally present in the liver and known as ciliated hepatic foregut cysts [2]. However, CFCs of the gallbladder are extremely rare. Only 13 cases of CFC of the gallbladder, including the present case, have been reported till date [1, 4–14] (Table 1). The median age was 39 years, with a range of 9–72 years. Ten of the thirteen patients were female. The most frequent clinical symptom was abdominal pain, seen in nine cases. The most frequent location was the neck, which was reported in nine cases, followed by the body in three cases. The median size of the cysts was 27 mm, with a range of 7–45 mm. Ten out of the 13 cases had a cyst with mucous content. All cases, including the present case, were pathologically diagnosed with gallbladder CFC.

It is often difficult to radiographically distinguish between benign and malignant CFCs. Most cases of gallbladder CFCs reported anechoic features on US and nonenhanced findings on CT [1, 4–14] (Table 1). In the present case, CT revealed a contrast-enhanced lesion with an unclear border along the liver, suggesting the possibility of malignancy. In MRI, gallbladder CFCs frequently show hyperintensity in T1 and T2 sequences, but sometimes, these appear isointense or hypointense in T1 sequences, which can be helpful in the diagnosis of CFCs of the gallbladder [15]. Furthermore, Han et al. [11] reported that MRI was superior to other modalities in diagnosing cystic lesions. In the present case, malignancy could have been ruled out if the lesion could be evaluated using MRI; however, MRI was not performed due to the patient's claustrophobia. Therefore, MRI may allow differentiation between benign from malignant CFCs of the gallbladder, especially when the possibility of malignancy cannot be ruled out.

No consensus has been reached regarding whether gallbladder CFCs should be resected in clinical practice. For gallbladder CFCs, some researchers insist that close surveillance is recommended when the cyst is asymptomatic [7], while others suggest excision should be performed due to its tendency for malignant transformation [4, 13, 14]. The biological behavior of CFCs of the gallbladder is not yet understood because of CFCs' rarity, and malignant CFCs of the gallbladder have not been reported to date [1, 4–14] (Table 1). However, malignancy can arise in hepatic CFCs [2, 16–18]. Bishop et al. [17] reported that 5.6% of cases have histological evidence of squamous cell carcinoma in hepatic CFCs. Due to the histological similarity between these two types of CFCs, the possibility of similar changes in gallbladder CFCs should be considered. Additionally, nine out of 13 cases of gallbladder CFCs in the literature search presented with abdominal pain [1, 4–14] (Table 1). Therefore, surgical excision is needed to rule out benign or malignant lesions and resolve patients' symptoms.

It remains to be clarified whether open surgery or laparoscopic surgery is the best procedure for CFCs of the gallbladder, although a recent guideline for biliary tract cancers recommended laparotomy for patients with suspected gallbladder carcinoma [19]. A literature search [1, 4–14] (Table 1) revealed that the most frequent surgical procedure was laparoscopic surgery, which was performed in 6 cases, followed by open surgery. Therefore, open surgery may be a better surgical procedure if malignant CFS is suspected, and laparoscopy if symptomatic but benign CFC is suspected. In recent years, single-port laparoscopic cholecystectomy has become popular due to its superior cosmetic appearance [20]. The surgical procedure may be indicated in CFC cases, which are relatively common in young women. Further investigation is needed to identify the appropriate procedure for CFCs of the gallbladder.

## 4. Conclusions

This report describes an extremely rare case of gallbladder CFC suspected of gallbladder cancer preoperatively. The present findings suggest that gallbladder CFCs can be considered as cysts requiring treatment due to their potential for malignant transformation and high-frequency symptoms.

## Figures and Tables

**Table 1:** Previous reports of the ciliated foregut cyst of the gallbladder.

No.	Author	Year	Age	Sex	Symptoms	Location	Size (mm)	Locularity	Cyst content	Image findings	Therapy	Malignancy
1	Nam^1)^	2000	36	F	Fever, vomiting, pruritic skin rash	Fundus	15	Unilocular	Mucus	US; anechoic/CT; homogeneous low density	Laparoscopy	No
2	Hirono^4)^	2002	43	F	No	Neck	25	Unilocular	Mucus	US/CT/MRI; cystic lesion with polyps	Open	No
3	Muraoka^5)^	2003	37	F	No	Body	24	Unilocular	Mucus	US; anechoic with highly echoic area/CT; nonenhanced lesion	Open	No
4	Bulut^6)^	2010	41	F	RUQ pain	Neck	35	Unilocular	Mucus	Unspecified	Laparoscopy	No
5	Tunçyürek^7)^	2013	42	F	RUQ pain	Body	7	Unilocular	Mucus	US; anechoic with highly echoic area	Laparoscopy	No
6	Giakoustidis^8)^	2014	29	F	Epigastric pain	Neck	30	Unilocular	Mucus	US; anechoic with solid elements/MRI; cyst adjacent to gallbladder	Laparoscopy	No
7	Hwang^9)^	2015	39	F	RUQ pain	Neck	35	Unilocular	Mucus	US/CT; unilocular cystic lesion with amorphous debris level	Laparoscopy	No
8	Lee^10)^	2015	61	M	RUQ pain	Body	27	Unilocular	Gelatinous	Unspecified	Laparoscopy	No
9	Han^11)^	2016	20	F	RUQ pain	Neck	16	Unilocular	Mucus	US/CT; nonenhanced cystic mass	Unspecified	No
10	Agarwal^12)^	2016	9	M	RUQ pain, vomiting	Neck	30	Unilocular	Mucus	Unspecified	Unspecified	No
11	Farrugia^13)^	2017	72	M	RUQ pain, nausea	Neck	45	Unilocular	Unspecified	US; anechoic/MRI; cyst adjacent to gallbladder	Open	No
12	Wissem^14)^	2017	34	F	RUQ pain	Neck	30	Unilocular	Mucus	CT; cyst adjacent to gallbladder	Open	No
13	Present case	2021	50	F	No	Neck	17	Unilocular	Mucus	US; isoechoic/CT; contrast-enhanced solid lesion	Open	No

RUQ: right upper quadrant; US: ultrasound; CT: computed tomography; MRI: magnetic resonance imaging.

## Data Availability

The [DATA TYPE] data used to support the findings of this study may be released upon application to the [DATA ACCESS COMMITTEE NAME or INSTITUTIONAL REVIEW BOARD NAME], who can be contacted at [CONTACT DETAILS].

## Abbreviations

CFCs: Ciliated foregut cysts
US: Ultrasonography
CT: Computed tomography
MRI: Magnetic resonance imaging.
